# Effects of Mine Tailings Exposure on Early Life Stages of Atlantic Cod

**DOI:** 10.1002/etc.4415

**Published:** 2019-06-20

**Authors:** Helena C. Reinardy, Kristine B. Pedersen, Jasmine Nahrgang, Marianne Frantzen

**Affiliations:** ^1^ Department of Arctic Technology University Centre in Svalbard Longyearbyen Svalbard Norway; ^2^ Akvaplan‐niva, Fram Centre Tromsø Norway; ^3^ Department of Arctic and Marine Biology UiT The Arctic University of Norway Tromsø Norway

**Keywords:** Cod, Embryos and larvae, Norway, Mine tailings, Copper, Gene transcription

## Abstract

In Norway, mine tailings waste can be deposited by coastal submarine dispersal. Mine tailings slurry includes fine particles <10 µm with elevated levels of metals (e.g., copper, iron) from residual mineral ore. Prolonged suspension of small particles in the water column may bring them into contact with locally spawned pelagic fish eggs, including Atlantic cod, *Gadus morhua*. Newly fertilized cod embryos were exposed to suspended mine tailings particles up to 3.2 mg/L in flow‐through aquaria for a total of 21 d. Significantly more particles adhered to the surface of the chorion from the high treatment after 11‐d exposure, and dissolved Cu concentrations increased in the water (up to 0.36 ± 0.06 µg/L). There was no adverse effect on embryo mortality but an 8% elevation in larval mortality. There were no differences with treatment on timing of hatching, embryo and larva morphometrics, abnormalities, or cardiac activity. There was a treatment‐dependent up‐regulation of stress marker genes (*hspa8*, *cyp1c1*) but no indication of metal‐induced activation of metallothionien (*mt* gene transcription). Transcription markers for DNA and histone methyltransferases did show treatment‐related up‐regulation, indicative of altered methylation in larvae when developmental methylation patterns are determined, indicating some level of chronic toxicity that may have longer‐term effects. *Environ Toxicol Chem* 2019;38:1446–1454. © 2019 The Authors. Environmental Toxicology and Chemistry published by Wiley Periodicals, Inc. on behalf of SETAC.

## Graphical abstract

Exposure to mine tailings particles increases mortality in Atlantic cod larvae and results in increased expression of genes involved in epigenetic regulation.

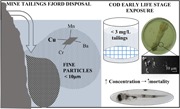

## INTRODUCTION

Mine tailings constitute the major waste product from extractive mineral industries and are made up of a fine‐grained slurry of milled rock, process chemicals, and elevated concentrations of metals from residual ore minerals (Kvassnes and Iversen 2013). In Norway, there are presently 7 active sites where mine tailings are disposed of through submarine pipes into fjords, and tailings can remain suspended for prolonged periods in the water column because of slow settling rates of small particles and can be traced (up to 10 km) by increased water turbidity over considerable distances from the deposit outlet (Berge et al. 2011; Ramirez‐Llodra et al. 2015). The tailings plume, and potential resuspension by currents, upwelling, or internal waves, can pose a risk to the pelagic ecosystem; however, studies on effects of mine tailings on marine fish are sparse (Olsvik et al. 2015a). In 2015, the mining company Nussir ASA was awarded a waste discharge permit in Repparfjorden, Finnmark, northern Norway, with an allowed discharge limit of 2 million tons of tailings per year; and mining activities are expected to begin in 2019. Tailings from the Nussir mine are rich in copper (Cu) but also contain other metals such as barium (Ba), nickel (Ni), and chromium (Cr) that may be released into the water (Pedersen et al. 2017). Environmental investigations of tailings deposited in Repparfjorden from when the mine was active in the 1970s showed dispersion of Cu from the disposal area to the middle part of the fjord, as a result of both tailings particle dispersion and desorption and transport of dissolved Cu (Pedersen et al. 2017).

Atlantic cod (*Gadus morhua*) from both coastal and northeast Arctic populations spawn in Norwegian fjords and are a major concern because the mine tailings may interact with and affect the early life stages (embryos and larvae) that coexist in and around tailings deposit sites. Atlantic cod spawn pelagic eggs in the range of 40–200 m depth in the period of March to May, and embryos, larvae, and juveniles persist in fjords for months or years depending on original stock and local water currents, with the potential for prolonged exposure to fine fraction mine tailings (Myksvoll et al. 2011). Studies on effects of suspended sediment and particle exposure on marine fish have shown that effects are a function of both exposure time and particle concentration. Increasing exposure time results in effects at lower particle concentrations (Chapman et al. 2014), and sublethal exposure can result in changes in habitat preference, avoidance and foraging behavior, growth, and recruitment and fish stock structure (Page 2014a, 2014b). Mine tailings from metal ore extraction can have additional metal and process chemical toxicity effects (Swedmark and Granmo 1981; Olsvik et al. 2015b), making evaluation of mine tailings effects a complex multistressor toxicological issue.

Understanding effects of environmental contaminants on early life stages of fish can inform on wider ecological implications because of their greater sensitivity and their future impacts on stock populations. Fish early life stages (embryos and larvae) are particularly vulnerable to environmental contaminants, primarily because of their rapid growth, underdeveloped organ systems including permeable external surfaces such as skin and gills, and small size that increases uptake into internal tissues (Bentivegna and Piatkowski 1998; Mohammed 2013). Rapid growth rates of embryos and larval fish can lead to high mortality, embryonic malformations, or erroneous development if the physical, biochemical, or nutritional requirements are not met (Lall and Lewis‐McCrea 2007). Sublethal effects on the genome and epigenome can potentially lead to long‐term transgenerational effects, genetic specialization, and divergence of stock structure (Jha 2004; Head 2014). Cod increase in size by more than 4000 times during the first 2 mo after hatching, and larvae predominantly eat copepod nauplii (Van der Meeren and Næss 1993), filtering particles down to 6 μm spherical diameter (Van der Meeren 1991). There is a likelihood for cod larvae to come into direct contact with mine tailing particles and elevated dissolved metals if the spawning site is in the area of mine tailings disposal.

The impact of release of mine tailings into fjord environments containing important spawning grounds of cod is still unknown. The present study investigated the effects of exposure to mine tailings sourced from the Nussir ASA copper mine site in Repparfjorden on early life stages of cod. Effects of particle and metal toxicity were analyzed by a suite of developmental, molecular, and chemical endpoints. It is critical to evaluate environmental contaminants for potential long‐term effects on organisms and changes that can be transmitted to the next generation mediated via impacted genetic and epigenetic mechanisms. Integration of epigenetic and genetic endpoints into ecotoxicological studies can inform on long‐term transgenerational effects, particularly for sublethal and environmentally relevant exposure scenarios (Jha 2004; Vandegehuchte and Janssen 2011). In addition, methylation patterns are determined early in development (Martin et al. 1999), and affected methylation of early life stages of fish can have implications on development and survival (Dorts et al. 2016). The results are aimed at understanding possible effects of resumption of mining and tailings deposition in Repparfjorden and potential impacts on key components of the local pelagic ecosystem such as early life stages of cod.

## METHODS

### Mine tailings exposure

Rock was provided by Nussir ASA and was made up of ore from the Nussir (90%) and Ulverryggen (10%) sites from Repparfjorden (Ramirez‐Llodra et al. 2015), Finnmark, Norway (70º28N, 24º13E). The rock was processed at SGS Mineral Services, Canada, to simulate the commercial mining metal extraction processes; and the produced mine tailings were mixed into 50 L concentrated slurry for the cod exposure experiment at the Akvaplan‐niva research facility at Kraknes, Tromsø, Norway. The slurry was made up daily and bubbled in a conical 100‐L tank to keep particles in suspension. A multichannel peristaltic pump (Watson Marlow 205U) delivered a constant flow (155 mL/h) of concentrated tailings into 3 header tanks via 1 tube (low treatment), 3 tubes (medium treatment), or 10 tubes (high treatment). A fourth header tank (control) was not supplemented with slurry. The header tanks were filled at a constant flow rate with flowing 60 µm filtered seawater, and gravity‐fed outflow was controlled by flow meters to deliver flow rates of approximately 6 to 10 L/h into triplicate flow‐through 6‐L fish aquaria at nominal tailings concentrations of 0 (control), 2 (low), 6 (medium), and 20 (high) mg tailings/L. Estimations of actual tailings concentration (mg/L) were calculated from a standard dilution curve (taking into account particle settlement in the slurry tank): y=ax+b, where *y* is the particle counts per mL, *a* is the slope of the regression, and *b* is the time‐matched control background particle counts, applied to daily aquaria particle concentrations (after additional particle settlement in header tanks) over a 21‐d exposure. Particle analyses (concentrations and size measurement) were conducted using a Beckman Multisizer 4 Analyzer, A39152, with a particle size profile detection range of 2 to 60 µm. Each aquarium was supplied with 3 suspended passive samplers (diffusive gradient in thin film [DGT]; Eurofins) at the start of the exposure: the first was removed after 8 d, the second after 15 d, and the third after 21 d of exposure for analysis of accumulated dissolved Cu. The water in each aquarium was measured daily for temperature and dissolved oxygen (OxiGuard); aquaria outflow water was sampled every day for particle analyses and weekly for water analysis (inductively coupled plasma optical emissions spectrometry [ICP‐OES]).

### Fish

All experimental work conformed to institutional and national regulations on the ethical use of fish in research, under the nonpermit guidelines for pre‐first‐feeding fish stages, according to the Norwegian Food Safety Authority.

Adult brood stock of Atlantic cod (*G. morhua*) were stripped, and the eggs and sperm from 4 females and 3 males were mixed and fertilized at the Nofima Centre for Marine Aquaculture, Tromsø, Norway, according to routine industry practice. The eggs were then allowed to settle and float for 2 h (to allow dead and unfertilized eggs to sink), and 10 mL of the buoyant fraction (approximately 5000 newly fertilized embryos, estimated by packed volume in seawater) were placed into the top compartment of each aquarium to initiate the exposure experiment. A mesh aquarium divider provided 6 L of water circulation, with embryos restricted to the top 3 L for ease of collection and observation, with constant gentle bubbling of air. The air bubbling was halted to allow live buoyant embryos to rise to the surface and dead embryos to sink to the mesh for daily detritus removal and mortality counts. Hatching was between days 14 and 17 and was estimated from photographs of the surface of the aquaria in addition to collection and counting of empty chorions. Chorions were collected daily and stored at –20 °C for metal analysis (ICP‐OES). Mortality (embryo, larval, and total) and hatching were calculated from arcsine‐transformed percentages based on retrospective day 4 total counts and tested by one‐way analysis of variance (ANOVA; or Kruskal‐Wallis for nonparametric data) and post hoc multiple range tests. Severe abnormalities were assessed at the point of terminal sampling (18–20 d of exposure, 5 d posthatching [dph] larvae) from images of larvae collected into a shallow Petri dish and photographed against a clear white background (ensuring minimal contact, with many larvae visible from the same image, a single image per aquarium; *n* = 43–300 larvae visible and analyzed in images). Abnormalities from these top‐view images were characterized as larvae showing clearly crooked bodies. Embryo and larval developmental morphometrics and subtle abnormalities (i.e., yolk sac edema, craniofacial deformities) were analyzed by subsampling (exposure days 7, 11 [embryos], 14–16 [1 dph], and 18–20 [5 dph] larvae). Subsamples of *n* = 13–43 (mean = 24 individuals/aquarium) were photographed under a stereo dissection microscope (Leica M205 C with a Leica MC170 HD camera), and morphometric measurements were carried out on individual images selected to matching orientation (e.g., side view for embryo total length and percent yolk, top view for head width and eye measurements) with ImageJ software (ImageJ 1.50i) with the scale set by a calibrator slide (WILD 310345). Total and spine length were calculated from whole individual images, and yolk sac, yolk, eye, and head measurements were calculated from detailed side‐view images (except head width from the dorsal view). Heartbeat activity (*n* = 9–14 individuals/aquarium) were recorded after 11 d of exposure (embryo) and 18 to 20 d of exposure (5 dph larvae) by carefully transferring embryos and larvae into a convex glass microscope crucible (larvae were lightly anesthetized with 50 mg/L tricaine methanesulfonate; Finquel MS‐222) balanced within a dish containing water and ice to maintain cool conditions for 1 min of video recording. Heartbeats were counted from timed video sections with clear footage (excluding periods with too much movement or swimming out of view) to calculate beats per minute. A few embryos (*n* = 3–10) were collected from each aquarium on day 11 of exposure and fixed in 4% buffered formalin for scanning electron microscopy (SEM) for imaging of particles on the chorion surface. At terminal sampling (18–20 d of exposure [5 dph larvae]) remaining live larvae were counted for retrospective total counts, and subsamples were stored as dry pellets at –80 °C for mRNA transcription analysis (200–400 pooled larvae per aquarium) and at –20 °C for ICP‐OES metal analysis (remaining larvae approximately 600–3300).

### Chorion surface analysis (SEM)

Embryos stored in 4% buffered formalin were transferred to an overnight fixation solution of 2.5% glutaraldehyde and 4% formaldehyde, followed by 1.5‐h 1% osmium fixation. Fixatives were removed by a series of increasing ethanol concentration washes before critical point drying, mounting with carbon tape, and coating with carbon. The mounted chorions were scanned using a MonoCL4 Zeiss SEM. Particle concentration on the surface of chorions was calculated by visual counts from images and surface area using ImageJ software.

### Metal analysis (ICP‐OES)

Metal concentrations were analyzed in weekly water samples, chorion collections, and terminal sampling of all remaining larvae from each aquarium. Water samples were vacuum‐filtered through a 0.45‐µm cellulose nitrate membrane filter (Sartorius) to remove particles. Chorions and larvae were filtered and freeze‐dried to remove residual water, digested in 10 mL 1:1 HNO_3_ (autoclave 120 °C, 30 min), filtered through a 0.45‐µm cellulose nitrate membrane filter (Sartorius) to remove particles, and diluted to 25 mL with distilled water. Liquid samples were analyzed by ICP‐OES on a Varian 720‐ES in the Arctic Technology Centre at the Technical University of Denmark, following an internally developed routine procedure, with double determination of each sample. A 9‐point standard curve (0.02–10 ppm for trace elements and 2–100 ppm for aluminum, calcium [Ca], iron [Fe], potassium [K], magnesium [Mg], and sodium) was made from certified reference wastewater (VKI Water Quality Institute, QC HL1, Cu concentration 1.99 ppm). Two of the standard curve dilutions were run repeatedly as controls after every 18 measurements. Calculated reference control samples for Cu varied 100 to 106%; the 0.5‐ppm Cu dilution sample was analyzed to be 0.49 ± 0.01 mean ± standard deviation (SD; range = 0.47–0.50) and the 1‐ppm Cu dilution sample was analyzed to be 0.98 ± 0.02 mean ± SD (range = 0.94–1.00). An internal yttrium spike control was added to all samples and standards in 2% nitric acid to ensure low pH. The DGT passive sampler analysis for Cu was performed by Eurofins using standard EN ISO17294‐1/2.

### mRNA transcription analysis

Total RNA was extracted from pooled 5‐dph larvae, following the manufacturer's guidelines (RNeasy Mini kit for animal tissue; Qiagen). Pellets were initially homogenized by pestle in RLT buffer (Qiagen) with 2β‐mercaptoethanol and additional tissue breakup by passing through 25‐G needle, 15‐min DNase treatment (Qiagen). The RNA was eluted into RNase‐free water; RNA concentration was determined by nanodrop, and quality was assessed by 260/280 and 260/230 ratios (Nanodrop 2000 spectrophotometer; Thermo Scientific) and visualized on a 1.2% agarose gel. Complementary DNA (cDNA) was synthesized from 500 ng RNA by reverse transcription (High‐Capacity cDNA kit; Applied Biosystems).

Atlantic cod primers were either selected from the literature or designed and verified for the present study (Supplemental Data, Table S1). Lyophilized primers (Eurofins) were reconstituted with RNase‐free water, and differential transcription of selected genes was analyzed by quantitative reverse‐transcription polymerase chain reaction (PCR; StepOne Plus; Applied Biosystems) with a SYBR green detection system (SYBR Green PCR Master Mix; Applied Biosystems). Primer concentrations were optimized, and PCR efficiency was calculated (*E* = 10^(–1/slope)^) from cDNA from reverse‐transcribed RNA pooled equally from all samples. Control genes (*rpl4*, *elf1a*, and *gapdh*) were tested for nonsignificant effects of experimental mine tailings treatment (one‐way ANOVA); relative fold change of genes of interest was calculated following the efficiency‐adjusted delta‐delta‐Cq method (Pfaffl 2001), and geometric means of the fold change from the 3 control genes are reported.

### Statistical analysis

All statistics were carried out in Statgraphics Centurion XVII.I (Statpoint Technologies). Data were tested for normality and homogeneity of variance, and treatment‐related differences were tested by one‐way ANOVA or Kruskal‐Wallis test, for parametric or nonparametric data accordingly, followed by post hoc tests for difference. Percentage data were arcsine‐transformed before testing for treatment‐related effects.

## RESULTS

**Figure 1:**
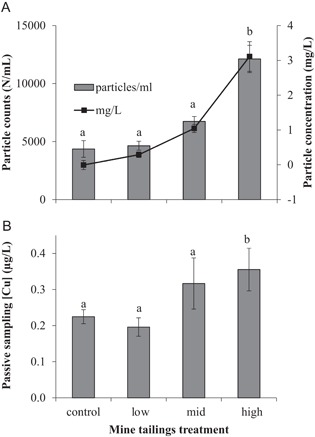
Exposure water conditions. (**A**) Particle counts and concentrations were significantly elevated in the high exposure treatment (generalized linear model, *F* ratio 16.68, *p* < 0.05, post hoc multiple comparisons 95% least significant difference, *n* = 20, 2‐ to 21‐d exposure, data are means ± standard deviation [SD]). (**B**) Dissolved Cu concentration was significantly elevated in the high treatment (letters indicate significant difference, Kruskal‐Wallis statistic 8.69, *p* < 0.05, post hoc multiple range tests, *n* = 3 aquaria after 21‐d exposure, data are mean ± SD).

Seawater oxygen levels in all aquaria remained >88% (mean 97.7% dissolved oxygen saturation) throughout the experiment period. Estimated tailings concentration in aquaria (mean ± SD, *n* = 20, 2–21 d of exposure) were 0 ± 0.02 (control), 0.36 ± 0.47 (low), 1.09 ± 0.88 (medium), and 3.23 ± 1.79 (high) mg tailings/L, with significantly elevated levels in the high treatment (generalized linear model [GLM]; *p* < 0.05, *F* ratio 16.68, post hoc least significant difference; Figure 1). Within the 2 to 60 µm detection range, the size of particles measured in the aquarium water ranged from 2 to 10 µm (2.5% particles >10 µm), and the high treatment contained slightly but significantly larger particles (control mean ± SD particle size 3.6 ± 0.35 µm, high treatment mean ± SD particle size 4.2 ± 0.27 SD µm; GLM, *p* < 0.05, *F* ratio 10.64, post hoc multiple comparisons 95% least significant difference, *n* = 20, 2–21 d of exposure). Because most of the particles were at the lower limit of size detection, small particles <2 µm are likely to be included in the tailings but were uncounted by this analysis.

Concentrations of Cu in the water were calculated by Cu accumulated on thin film passive samplers over the 22‐d exposure, and there was a significant increase in dissolved Cu in the high treatment (mean ± SD 0.36 ± 0.06 µg/L) compared with the control treatment (mean ± SD 0.22 ± 0.02 µg/L; Kruskal‐Wallis statistic 8.69, *p* < 0.05, post hoc multiple range tests; Figure 1B). Total Cu concentration in the water, as measured by ICP‐OES, ranged from 2.63 ± 1.27 to 4.70 ± 1.60 µg/L (mean ± SD, aquaria averages over 22‐d exposure, treatment averages Table 1) with no significant difference between treatments (Kruskal‐Wallis test statistic 1.51, *p* > 0.05). Selected metals analyzed in the collected hatched chorions and larvae were significantly elevated with respect to the treatment levels, including Cu, Ba, Cr, Mg, Mn, K, zinc, vanadium, Ca, cobalt, and Fe; however some data sets contained samples that were below detection limit and therefore had reduced technical replicates (Table 1; Supplemental Data, Table S2).

**Table 1:** Cu concentrations analysed by ICP‐OES in exposure water (averaged from weekly samples over 22 d exposure), hatched chorions (total collected between days 13 and 19), and cod larvae (sampled 5 d after peak hatch, 19 to 21 d exposure)^a^

Metal	Treatment	Water (µg/L)	Chorions (mg/kg)	Larvae (mg/kg)
Cu	Control	4.27 ± 0.33	0.75 ± 0.21A^b^	0.85 ± 0.46
	Low	3.86 ± 0.94	0.71 ± 0.08A	0.59 ± 0.29
	Medium	3.48 ± 0.97	1.75 ± 0.38A	0.60 ± 0.11
	High	3.42 ± 0.80	3.198 ± 1.04B	0.52 ± 0.08

^a^ Uppercase letters indicate significant difference (one‐way analysis of variance, post hoc multiple range test). Data are means ± standard deviation, n = 3 aquaria per treatment.

^b^ Significant regression (simple regression, *p* < 0.05, *r* ^2^ = 80.3%).

**Figure 2:**
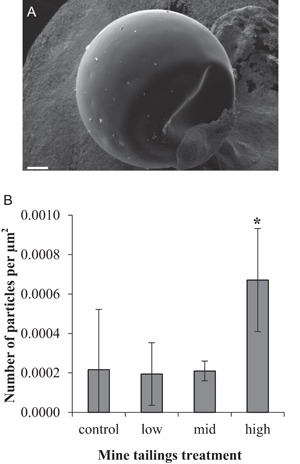
Particle concentration on chorion surface. (**A**) Example scanning electron micrograph image of whole chorion (11‐d exposure) showing presence of particles on surface. Scale bar = 100 µm. (**B**) Particle counts from micrograph images (data are means ± standard deviation [SD], *n* = 3–9 embryos per treatment). *Significantly higher particle concentration on surface of chorion from high treatment (one‐way analysis of variance, *F* ratio 3.41, post hoc multiple range tests).

Increased number of mine tailings particles in the water corresponded with an increased number of particles adhered to the surface of cod chorion after 11‐d exposure (one‐way ANOVA, *F* ratio 3.41, post hoc multiple range tests; Figure 2). The adhered particles were observed to range from 0.5 to 10 µm in size, similar to the analyzed size profile of mine tailings between 2 and 10 µm (with a lower detection limit of 2 µm).

**Figure 3:**
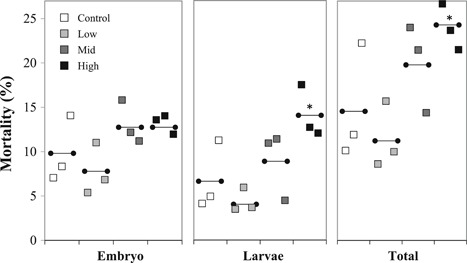
Mortality of early life stages of cod. Embryo (days 4–15), larvae (days 15–19), and total (days 4–19) mortality. Data squares are values in each aquarium (*n* = 3 aquaria per treatment, horizontal lines indicate treatment means). *Significantly different from controls (one‐way analysis of variance on arcsine‐transformed percentage data, *F* ratio 5.16, post hoc multiple range tests).

Final retrospective counts resulted in an average of 4890 embryos/aquarium (range = 3685–7669 embryos), and there was no significant difference in total embryo numbers with respect to treatment level (Kruskal‐Wallis statistic 0.44, *p* > 0.05, *n* = 12 aquaria). There were relatively high levels of natural mortality in the first 4 d of exposure from unsuccessful development of early cell stages and unfertilized eggs still sinking out, unrelated to exposure treatment; and therefore exposure‐related mortality was analyzed from day 5 onward. The timing of hatching between the aquaria was variable and spread over 4 d (except for the low treatment where peak hatching occurred on a single day); there was no significant effect of treatment on hatching (GLM, *F* ratio 22.54, *p* > 0.05; Supplemental Data, Figure S1), but there was a significant correlation between peak hatching day and density of total embryos in the aquaria (simple regression, *p* < 0.05, *r* ^2^ = 61.2), with the highest‐density aquaria hatching first. There was no significant difference in embryo mortality between treatments (one‐way ANOVA, *F* ratio 2.84, *p* > 0.05), but after hatching the high treatment group had 8% higher mortality (mean ± SD 14.1 ± 3.0 %, *n* = 3 aquaria) compared with the control treatment group (mean ± SD 6.8 ± 2.3 %, *n* = 3 aquaria), resulting in an overall higher total mortality in the high treatment aquaria over the whole 22‐d exposure period (one‐way ANOVA, *F* ratio 4.17, *p* < 0.05; Figure 3). Morphometric analyses indicated no overall effect of exposure on developing embryos and larvae, including no significant differences in length, yolk absorption, abnormalities, and heart rate (*p* > 0.05, one‐way ANOVA for normally distributed data or Kruskal‐Wallis for nonparametric data; Supplemental Data, Table S3).

**Figure 4:**
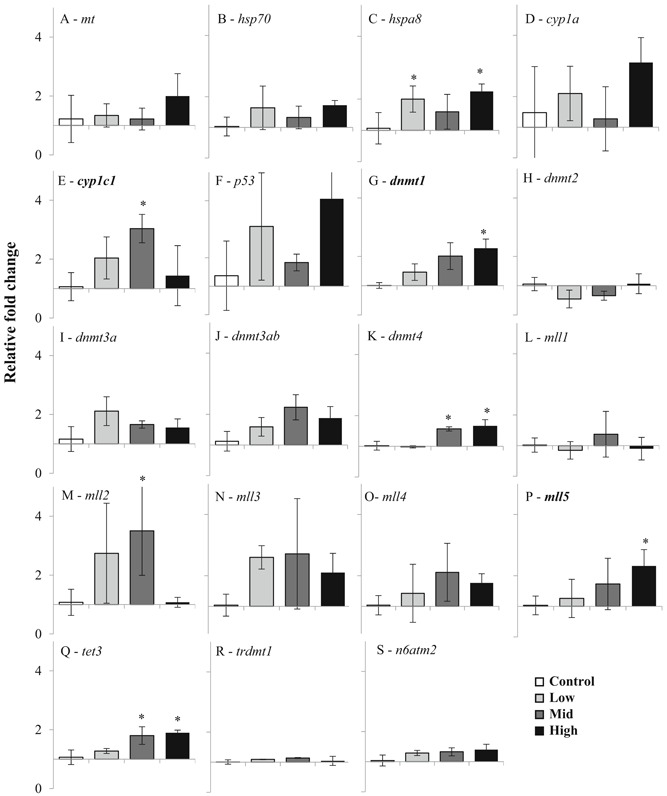
Gene transcription analysis (quantitative real time‐polymerase chain reaction) in larvae. Up‐regulation of genes involved in general stress and metal toxicity response (**A–F**) and epigenetic regulation marker genes (**G–S**) involved in methylation of DNA and histones from pooled larvae (approximately 200–400 larvae/aquarium). Data are means ± standard deviation, *n* = 3 aquaria per treatment, efficiency‐adjusted relative fold change (Pfaffl 2001). *Significantly different from control (one‐way analysis of variance and post hoc multiple range test); bold gene symbol indicates significant concentration‐dependent induction (simple regression, *p* < 0.05).

Gene transcription analyses were carried out on pooled larvae sampled at the termination of the experiment (5 dph). A panel of general stress (*hsp70*, *hsp8*, *cyp1a*, *cyp1c1*, and *p53*) and metal detoxification (metallothionein [*mt*]) marker genes showed consistent trends in treatment‐dependent up‐regulation, including significant (one‐way ANOVA and post hoc multiple range test) up‐regulation of *hspa8* (high treatment mean ± SD 2.2 ± 0.2–fold change) and *cyp1c1* (medium treatment mean ± SD 3.0 ± 0.5, high treatment 1.4 ± 1.0–fold change; Figure 4) but nonsignificant up‐regulation in the high treatment of *p53* (mean ± SD 4.1 ± 2.6–fold change) and *mt* (mean ± SD 1.2 ± 0.5–fold change). A wide panel of epigenetic gene markers showed consistent treatment‐dependent up‐regulation, significant (one‐way ANOVA and post hoc multiple range test) for *dnmt1*, *dnmt4*, *mll2*, *mll5*, and *tet3* (Figure 4).

## DISCUSSION

Submarine disposal of mine tailings in coastal areas involves a main discharge plume that rapidly sinks and settles within the area designated for disposal and a diffuse plume of smaller particles that remain suspended in the water column (Ramirez‐Llodra et al. 2015). The mining permit for Nussir ASA limits concentrations of mine tailings particles in the water column (above 30 m depth) at 3 mg/L, 1 km from the point of discharge. As such, the particle concentrations in the present study (up to 3.23 mg/L) represent accepted mine tailings concentrations in the pelagic zone of the defined area influenced by the tailings deposition. Fjord environments can fluctuate in background concentrations of suspended sediment depending on seasonal blooms, riverine input, and glacial melt (Syvitski et al. 1987), and concentrations can exceed 1000 mg/L (Farrow et al. 1983), with sedimentation peaking at approximately the late spring bloom (Wassmann et al. 1996). The particle concentrations in the present study do not reflect environmental concentrations beyond background fluctuations; however, the composition of the mine tailings differs from the naturally occurring sediment particles primarily in particle shape, organic and metal content, and associated biofilm (Ramirez‐Llodra et al. 2015; Pedersen et al. 2018). Particles were found to adhere to the surface of the chorions around the embryos, with significantly more on the embryos from the high exposure treatment, which suggests that they are mainly mine tailings particles. The chorion acts as a protective shield around the developing embryo and allows for gas exchange through the porous shell (Davenport and Lönning 1980). In the present study there was no evidence of particles clogging or blocking the chorion pores, and that there were no significant effects on embryo size, cardiac activity, morphometrics, timing of hatching, or mortality indicates that gas exchange was not affected. It is known that exposure to suspended sediments can reduce the buoyancy of cod embryos (Westerberg et al. 1996; Fehmarn Belt Environmental Consortium 2013), but it remains unknown whether the particles that adhered to the chorion affected buoyancy in the present study.

Increased mortality occurred in the larval stages after hatching out of the protective barrier of the chorion and coming into direct contact with the particles, suggesting that posthatching is a particularly sensitive stage. This increase in larval mortality is likely attributable to combined effects of life stage, particle exposure, and differences in mine tailings particle size and shape characteristics compared with suspended sediment exposures (Westerberg et al. 1996; Hall et al. 2004). At approximately the period of hatching, somite development is complete and no functional gills or mouth parts are present (Hall et al. 2004). The 3‐ to 5‐d period after hatching may be a particularly sensitive time as the larva orientates within the water and mouth parts and jaw develop for first feeding (Hall et al. 2004). Other studies have found increased avoidance behavior in larval cod exposed to suspended sediment concentrations >3 mg/L (Westerberg et al. 1996) and >10 mg/L in adult cod (Fehmarn Belt Environmental Consortium 2013; Page 2014b) and no significant increase in mortality of larvae exposed to up to 1000 mg/L but with few experimental exposure details included (Fehmarn Belt Environmental Consortium 2013). Studies with older post‐first‐feeding larvae and juvenile cod exposed to concentrations of 20 to 130 mg/L suspended sediments showed signs of clogged gills and histopathological changes to delicate osmoregulatory surfaces, impeding oxygen transport (Lowe et al. 2015), indicating that later stages are also vulnerable but may differ in the mode of effects as body systems develop and grow.

The present study found significant increases in Cu concentrations in the water from desorption from the tailings, with a maximum concentration of 0.4 µg/L detected in a high treatment aquarium, as measured by the DGT passive samplers. The Cu concentration in the water, as measured by ICP‐OES, was an order of magnitude higher than that detected by passive sampling, in accordance with observations from other studies (Uher et al. 2018), and the treatment‐related differences were no longer significant. According to the ICP‐OES data, the concentration is within the class 4 (class orange, “bad,” 2.6–5.2 µg/L) range of the Norwegian coastal water quality standards, above the predicted‐no‐effect concentration but below adverse‐effect concentrations (Miljødirektoratet 2016), but still within the range typical of background coastal systems including seawater concentrations of Tromsø (Kramvik 2013). The Cu concentrations from water from Repparfjorden are an order of magnitude lower, ranging from 0.2 to 0.5 µg/L (Christensen et al. 2011). Copper has a high affinity for organic matter, and Cu associated with organic matter (dissolved organic carbon or organic ligand complexes), suspended solids, or colloids diffuses into the DGT passive samplers; therefore, DGT passive samplers are considered to reflect the bioavailable metal content (Fernandez‐Gomez et al. 2012). The passive samplers indicate concentrations of bioavailable Cu <0.4 µg/L, which places the quality of the water below class 2 (class green, “good”) classification (Miljødirektoratet 2016). The large surface area to volume ratio of small particles increases the likelihood of metal release (John and Leventhal 1995), and it can be estimated that 5 to 8% of the total Cu in the mine tailings particles dissolved into the water and accumulated in the DGT passive samplers in the high treatment aquaria over the period of the exposure. Physicochemical characterization of the same tailings estimated that up to 10% of Cu is loosely bound in an exchangeable fraction (Pedersen et al. 2017); therefore, the present results partly support this finding by measuring up to 8% bioavailable dissolved Cu in water. The elevated levels of Cu detected in the chorions is due to both Cu bound to particles adhered to the chorion surface, before hatching and immediately after hatching before the daily collection, as well as potentially dissolved Cu taken up into the chorion. However, there was no significant elevation in total Cu concentrations in the larvae, which suggests that any slight increase in concentrations in water and chorions was not elevated enough for bioaccumulation into embryos and larvae. This is also supported by no significant elevation of metallothionein gene transcription in hatched larvae, a sensitive marker for induction of metal detoxification mechanisms (Dorts et al. 2016). Previous studies on cod embryos and larvae found elevated mortality in larvae exposed to nominal concentrations of 11.5 μg/L Cu (Granmo et al. 2002) and prolonged developmental time and morphological abnormalities including spinal deformations, decreased hatching rate, and reduced larval viability after exposure to nominal Cu concentrations of approximately 10 μg/L (Swedmark and Granmo 1981). It is highly unlikely that the increased larval mortality in the present study was due to metal toxicity from exposure concentrations below expected toxic levels, with maximum water concentrations of 0.4 μg/L bioavailable Cu and no indication of bioaccumulation.

Gene transcription analyses can be a sensitive tool for investigating sublethal effects in environmentally relevant exposure experiments. Integration of potentially heritable endpoints such as epigenetics into ecotoxicological studies can inform on long‐term transgenerational effects, particularly for sublethal chronic exposure scenarios (Vandegehuchte and Janssen 2011; Head 2014); and the present study included a selection of epigenetic marker genes to investigate the potential for long‐term heritable changes in epigenome stability and control. There was a consistent pattern of up‐regulation of general stress marker genes (heat shock proteins and cytochrome P450 genes), including significant up‐regulation of *hspa8* and *cyp1c1*. These results suggest a sublethal detoxification response occurring in the larvae under exposure to mine tailings. Oxidative stress can be induced by particle exposure (Li et al. 2003) and has been linked to control of DNA methylation (Olsvik et al. 2015a). A previous study demonstrated an inverse relationship in up‐regulation of transcription of oxidative stress genes and down‐regulation of DNA methyltransferase gene transcription (Olsvik et al. 2015a). It is unclear what the biological consequences of down‐regulation (Olsvik et al. 2015a) compared with up‐regulation (the present study) of methylation control may be, but significant differences from unexposed controls suggest that methylation control mechanisms are affected by exposure. Methylation patterns of DNA and histones are set in early development (Martin et al. 1999), and affected methylation of early life stages of fish can have implications on development and survival (Dorts et al. 2016), including in cod (Skjærven et al. 2014). Both DNA methyltransferases (e.g., *dnmt* genes) and histone methyltransferases (*mll* genes) regulate transcription and expression of other genes and control patterns of methylation and demethylation in cod (Skjærven et al. 2014; Olsvik et al. 2015a). Results indicate sublethal mechanisms of dysregulation of methylation patterns after exposure to Cu‐rich mine tailings in cod early life stages, but the long‐term impact on development, survival, and population levels remains unknown. Both acute and chronic toxicological effects detected in early life stages have the potential for ecological impacts on spawning cod populations in areas of mine tailings disposal, and further eco‐ and toxicological studies are required to understand the impacts of submarine mine tailings disposal in locations overlapping areas used by cod for spawning.

## Data Accessibility

Data associated with the present study can be accessed on request to the author (helena.reinardy@sams.ac.uk).

## Supporting information

This article contains online‐only Supplemental Data.

Supporting information
