# Supplementary material for: Effects of Mine Tailings Exposure on Early Life Stages of Atlantic Cod
Source: Environ Toxicol Chem. 2019 Jun 20;38(7):1446–54. doi: 10.1002/etc.4415 (PMC6851963; doi:10.1002/etc.4415)
Supplement: Supplementary file 1 — Supporting information [file ETC-38-1446-s001.docx]

**SUPPLEMENTARY INFORMATION**

**Acute and chronic effects of mine tailings on early life stages of Atlantic cod**

Helena C. Reinardy, Kristine B Pedersen, Jasmine Nahrgang, Marianne Frantzen

Pages S1-S7

**Table S1**: Gene specific primers for Atlantic cod (*Gadus morhua*) for general stress and epigenetic markers. ^a^Gene identifiers from National Centre for Biotechnology Information.

**Table S2**: Metal concentrations in exposure water (**ICP-OES,** averaged from weekly samples over 22 d exposure), hatched chorions (total collected between days 13 and 19), and cod larvae (sampled 5 days after peak hatch, 19-21 d exposure). Data are means ± s.e.m., n=3 aquaria per treatment except where specified,*significantly different from control (One-way ANOVA, *post-hoc* multiple range test), *significant regression* (simple regression, p < 0.05).

**Table S3:** Morphometric analyses of development of cod embryos and larvae. Data are means ± s.e.m., n=3 aquaria per treatment,*significantly different from control (one-way ANOVA p<0.05, *post-hoc* multiple range test), larvae days post hatching (dph). Mine tailings treatment levels were 0 mg/l mine tailings (control), 0.36 mg/l (low), 1.06 mg/l (medium), and 3.23 mg/l (high).

**Figure S1:** Timing of larval hatching. Data are percentage means ± s.e.m., n=3 aquaria per treatment. Modelled with 4 parameter logistic regression ($y=y_{0}+ \frac{a}{1+ \left( \frac{x}{x_{0}} \right)^{b}}$), p < 0.05 model fit; no difference between models from different treatments, GLM F-ratio 141.87, p > 0.05.

**References**

**Table S1:** Gene specific primers for Atlantic cod (*Gadus morhua*) for general stress and epigenetic markers. ^a^Gene identifiers from National Centre for Biotechnology Information.

| **Gene** | **Gene identifier^a^** | | | **Forward primer (5’-3’)** | **Reverse primer (5’-3’)** | **Product size (bp)** | | **Reference** |
| --- | --- | --- | --- | --- | --- | --- | --- | --- |
| **Stress markers** | | | | | |  | |  |
| *mt* | | | CO542775 | CCTTGCGACTGCACCAAGA | CAGTTTAGGCAGGTGCATGATG | 63 | | Olsvik et al. 2015 |
| *p53* | | | EX723548 | CGCTGCTGCTGAACTTCATG | GGATGGCTCTCCGGTTCAT | 63 | | Olsvik et al. 2015 |
| *cyp1a1* | | | EX725014 | CCTTGACCTCTCGGAGAAAGAC | CGCCCCGCTAGCTATAGACA | 149 | | Olsvik et al. 2015 |
| *cyp1c1* | | | GmE100215i311891.909 | GTCAACCAGTCGTCCGTGAA | CGTTGTTCGTGAGGTCCTTGT | 115 | | Holen & Olsvik 2016 |
| *hsp70* | | | JF433917 | TCCGCATCATCAACGAACCA | AAGATGAGTGTCACCAGCGG | 176 | | Caipang et al. 2011 |
| *hspa8* | | | EX741726 | CATGACATCGTCCTGGTTGGT | CGTAGGCCACAGCTTCATCA | 121 | | Olsvik et al. 2015 |
| **Epigenetic marker genes** | | | | | | | | |
| *dnmt1* | | isotig24703, isogroup18253 | | ACCGCTTCTTTGGCAACATT | ATCCGCTCCAAGATGCACTT | | 63 | Olsvik et al. 2015 |
| *dnmt2* | | gu475966 | | GCCAGGGATTCACTGCTAAA | CCAAGACTGGTAGGCGACAC | | 80 | Gianneto et al. 2013 |
| *dnmt3a* | | GmE090818r466 | | GGGCGACTGTTCTTCGAGTTC | TCCCATGGCAACAACGTTCT | | 105 | Olsvik et al. 2015 |
| *dnmt3ab* | | F5A5JTJ01AUULY | | CGACGATGGCTACCAGTCCTA | AGAAACACCGGCAGCAGTT | | 89 | Skjaeven et al. 2014 |
| *dnmt4* | | FCBCL5V01ES7A4 | | GGTTCGGAGACGGCAAGTT | TGTACGAGGACGGGTTGAAGA | | 90 | Skjaeven et al. 2014 |
| *trdmt1* | | GmE090818c21047 | | CTTCCCAGACACGCCTCTCT | CAGCCCGATTCTCGTGAAAG | | 124 | Skjaeven et al. 2014 |
| *n6atm2* | | GmE100127i28226 | | GGAGTCGTCGAGGGTTCTGA | GCGCCAGTGGATGGTTGTA | | 112 | Skjaeven et al. 2014 |
| *tet3* | | ENSGMOG00000008079 | | ACAGGCAGACCCTTACAACG | AGTGCAGTGAGATTGGGGTG | | 123 | This study |
| *mll1* | | gu441836 | | GACCAGCCTAAGATCCAGAGCCA | GACAAGATCTTCTCCCGCTCCTC | | 179 | Nagasawa et al. 2012 |
| *mll2* | | gu441837 | | AGCAGAACCGTGGCATCTAC | CGAGTGATTTGCGTAGCGTG | | 95 | This study |
| *mll3a* | | gu441838 | | CGAGTACATCGGAACCATCA | ACGTACCTCGCAGGTCCTC | | 147 | Nagasawa et al. 2012 |
| *mll4* | | gu441839 | | AAGTACTCAGGTGCTGCCTC | TTGGGAGGGCTTTCTTCGTC | | 74 | This study |
| *mll5* | | gu441840 | | CATGAGCATAGGCATCCCAGT | CCTCCACTGATTCAGGTTCCG | | 89 | This study |
| **Control genes** | | | | | | |  |  |
| *rpl4* | EX725958 | | | GGTGCCATACAGCTGATCCA | CCAGGCATCACACTGCAGAA | | 126 | Olsvik et al. 2008 |
| *elf1a* | EX722124 | | | CGGTATCCTCAAGCCCAACA | GTCAGAGACTCGTGGTGCATCT | | 93 | Penglase et al. 2015 |
| *gapdh* | EX725566 | | | CCACGACAACTTTGGCATCGT | AGGGTCCGTCCACTGTCTTCT | | 83 | Modified from Olsvik et al. 2008 |

Table S2: Metal concentrations in exposure water (ICP-OES, averaged from weekly samples over 22 d exposure), hatched chorions (total collected between days 13 and 19), and cod larvae (sampled 5 days after peak hatch, 19-21 d exposure). Data are means ± s.e.m., n=3 aquaria per treatment except where specified,*significantly different from control (One-way ANOVA, *post-hoc* multiple range test), *significant regression* (simple regression, p < 0.05).

| **Metal** | **Treatment** | **Water** | **Chorions** | **Larvae** |
| --- | --- | --- | --- | --- |
|  |  | **[mg/L]** | **[mg/kg]** | **[mg/kg]** |
| **Ba** | **Control** | 0.0030 ± 0.0002 | ***0.187 ± 0.044*** | 0.073 ± 0.009 |
|  | **Low** | 0.0028 ± 0.0003 | ***0.153 ± 0.030*** | 0.048 ± 0.006 |
|  | **Mid** | 0.0029 ± 0.0002 | ***0.744 ± 0.164*** | 0.077 ± 0.012 |
|  | **High** | 0.0033 ± 0.0002 | ***1.797 ± 0.495**** | 0.079 ± 0.012 |
| **Cr** | **Control** | 0.0030 ± 0.0002 | ***0.219 ± 0.067*** | 0.213 ± 0.075 |
|  | **Low** | 0.0029 ± 0.0001 | ***0.210 ± 0.008*** | 0.075 ± 0.004* |
|  | **Mid** | 0.0027 ± 0.0002 | ***1.290 ± 0.460**** | 0.398 ± 0.132 |
|  | **High** | 0.0027 ± 0.0001 | ***1.347 ± 0.214**** | 0.250 ± 0.120 |
| **Al** | **Control** | 0.0064 ± 0.0002 | 40.36 ± 29.8 | 5.270 ± 2.315 |
|  | **Low** | 0.0060 ± 0.0008 | 11.43 ± 1.8 | 2.823 ± 0.412 |
|  | **Mid** | 0.0056 ± 0.0003 | 39.66 ± 7.4 | 4.926 ± 1.567 |
|  | **High** | 0.0064 ± 0.0003 | 80.20 ± 23.1* | 4.429 ± 1.046 |
| **Mg** | **Control** | 941.0 ± 65.3 | ***76.61 ± 223.6*** | 128.14 ± 36.68 |
|  | **Low** | 872.4 ± 118.4 | ***65.12 ± 12.2*** | 261.09 ± 21.69* |
|  | **Mid** | 939.9 ± 34.2 | ***135.88 ± 12.4*** | 126.86 ± 12.12 |
|  | **High** | 919.2 ± 61.1 | ***213.02 ± 51.7**** | 175.17 ± 26.87 |
| **Mn** | **Control** | ***0.0002 ± 0.0002*** | ***0.762 ± 0.04*** | ***0.176 ± 0.056*** |
|  | **Low** | ***0.0002 ± 0.0001*** | ***1.062 ± 0.25*** | ***0.105 ± 0.001*** |
|  | **Mid** | ***0.0001 ± 0.00002*** | ***7.574 ± 1.74*** | ***0.144 ± 0.016*** |
|  | **High** | ***0.0009 ± 0.0005***  ***n=2-3*** | ***18.839 ± 5.55**** | ***0.444 ± 0.178****  ***n=2-3*** |
| **K** | **Control** | 309.5 ± 23.1 | ***42.31 ± 14.1*** | 51.79 ± 10.42 |
|  | **Low** | 359.6 ± 39.8 | ***35.70 ± 4.2*** | 82.98 ± 14.07* |
|  | **Mid** | 380.0 ± 12.0 | ***61.50 ± 5.3*** | 42.45 ± 2.90 |
|  | **High** | 376.4 ± 18.6 | ***91.58 ± 15.4**** | 58.33 ± 7.16 |
| **Co** | **Control** | 0.0043 ± 0.0001 | 0.141 ± 0.044 | ***0.118 ± 0.023*** |
|  | **Low** | 0.0044 ± 0.0001 | 0.188 ± 0.036 | ***0.067 ± 0.019*** |
|  | **Mid** | 0.0044 ± 0.0001 | 0.140 ± 0.040 | ***0.119 ± 0.037*** |
|  | **High** | 0.0044 ± 0.0004 | 0.187 ± 0.042 | ***0.175 ± 0.013**** |
| **Pb** | **Control** | 0.026 ± 0.001 | 0.375 ± 0.063 | 0.584 ± 0247 |
|  | **Low** | 0.027 ± 0.002 | 0.256 ± 0.030 | 0.409 ± 0.265 |
|  | **Mid** | 0.027 ± 0.001 | 0.328 ± 0.042 | 0.281 ± 0.100 |
|  | **High** | 0.025 ± 0.002 | 0.292 ± 0.082 | 0.157 ± 0.067 |
| **Sb** | **Control** | 0.021 ± 0.001 | 0.512 ± 0.0064 | 0.594 ± 0.231 |
|  | **Low** | 0.017 ± 0.007 | 0.562 ± 00.466 | - |
|  | **Mid** | 0.026 ± 0.004 | - | 0.646 ± 0.300 |
|  | **High** | 0.025 ± 0.004 | 0.994 ± 0.781 | 0.079 |
|  |  |  | N=0-3 | N=0-3 |
| **Zn** | **Control** | 0.0043 ± 0.0012 | ***1.+65 ± 0.205*** | 5.022 ± 1.426 |
|  | **Low** | 0.0040 ± 0.0014 | ***1.970 ± 0.108*** | 4.923 ± 0.671 |
|  | **Mid** | 0.0025 ± 0.0005 | ***1.899 ± 0.160*** | 3.705 ± 0.326 |
|  | **High** | 0.0048 ± 0.0006 | ***2.418 ± 0.172*** | 4.437 ± 0.688 |
|  |  |  | Excl. cont. out |  |
| **As** | **Control** | 0.037 ± 0.0022 | 0.667 ± 0.317 | 0.578 ± 0.216 |
|  | **Low** | 0.034 ± 0.0016 | 0.669 ± 0.030 | 0.284 ± 0.032 |
|  | **Mid** | 0.025 ± 0.0005 | 0.577 ± 0.137 | 0.528 ± 0.016 |
|  | **High** | 0.031 ± 0.0031 | 0.611 ± 0.129 | 0.642 ± 0.058 |
| **V** | **Control** | 0.0015 ± 0.0000 | ***0.123 ± 0.040*** | 0.064 ± 0.017 |
|  | **Low** | 0.0014 ± 0.0002 | ***0.070 ± 0.021*** | 0.036 ± 0.012 |
|  | **Mid** | 0.0018 ± 0.0001 | ***0.181 ± 0.035*** | 0.049 ± 0.017 |
|  | **High** | 0.0015 ± 0.0001 | ***0.415 ± 0.115**** | 0.049 ± 0.005 |
| **Zr** | **Control** | 0.0003 ± 0.0002 | 0.087 ± 0.067 | - |
|  | **Low** | 0.0003 ± 0.0002 | 0.013 ± 0.004 | - |
|  | **Mid** | 0.0003 ± 0.0001 | - | - |
|  | **High** | 0.0001 ± 0.0000 | 0.016 | - |
|  |  |  | N=0-3 |  |
| **Ni** | **Control** | - | 0.410 ± 0.133 | 0.340 ± 0.150 |
|  | **Low** | - | 0.313 ± 0.003 | 0.112 ± 0.153 |
|  | **Mid** | - | 2.990 ± 1.373 | 0.999 ± 0.395 |
|  | **High** | - | 1.483 ± 0.772 | 0.581 ± 0.362 |
| **Ca** | **Control** | 276.1 ± 21.3 | ***331.8 ± 108.4*** | 346.7 ± 176.0 |
|  | **Low** | 255.6 ± 35.0 | ***217.4 ± 68.7*** | 422.2 ± 73.6 |
|  | **Mid** | 274.1 ± 10.1 | ***394.2 ± 83.7*** | 160.3 ± 9.1 |
|  | **High** | 268.7 ± 18.4 | ***660.3 ± 111.1**** | 310.5 ± 125.5 |
| **Cd** | **Control** | 0.0018 ± 0.0002 | 0.101 ± 0.020 | 0.088 ± 0.006 |
|  | **Low** | 0.0090 (n=1)* | 0.151 ± 0.004* | 0.047 ± 0.010* |
|  | **Mid** | 0.0013 ± 0.0001 | 0.115 ± 0.012 | 0.103 ± 0.015 |
|  | **High** | 0.0016 ± 0.0001 | 0.110 ± 0.011 | 0.092 ± 0.008 |
| **Fe** | **Control** | - | ***18.94 ± 7.2*** | 9.439 ± 2.305 |
|  | **Low** | - | ***12.29 ± 2.2*** | 3.327 ± 0.689* |
|  | **Mid** | - | ***49.43 ± 10.8*** | 4.525 ± 1.418 |
|  | **High** | - | ***105.69 ± 30.3**** | 4.063 ± 0.904 |
| **Sr** | **Control** | 0.500 ± 0.035 | 0.205 ± 0.083 | 0.677 ± 0.408 |
|  | **Low** | 0.454 ± 0.058 | 0.172 ± 0.062 | 0.849 ± 0.186 |
|  | **Mid** | 0.484 ± 0.014 | 0.177 ± 0.024 | 0.283 ± 0.037 |
|  | **High** | 0.477 ± 0.032 | 0.207 ± 0.036 | 0.592 ± 0.313 |

**Table S3:** Morphometric analyses of development of cod embryos and larvae. Data are means ± s.e.m., n=3 aquaria per treatment,*significantly different from control (one-way ANOVA, p < 0.05, *post-hoc* multiple range test), larvae days post hatching (dph). Mine tailings treatment levels were 0 mg/l mine tailings (control), 0.36 mg/l (low), 1.06 mg/l (medium), and 3.23 mg/l (high).

|  | **Mine tailings treatment** | **Developmental stage** | | | | | | |
| --- | --- | --- | --- | --- | --- | --- | --- | --- |
|  |  | **Embryo** | | | **Larvae** | | | |
|  |  |  | |  | 1 dph | | | 5 dph |
|  |  | **Exposure Day** | | | | | | |
|  |  | 7 | 11 | | | 14-16 | 18-20 | |
| **Chorion size** (mm^2^) | Control | 1.25 ± 0.02 | 1.26 ± 0.01 | | |  |  | |
|  | Low | 1.27 ± 0.02 | 1.25 ± 0.01 | | |  |  | |
|  | Medium | 1.27 ± 0.02 | 1.27 ± 0.01 | | |  |  | |
|  | High | 1.26 ± 0.01 | 1.27 ± 0.01 | | |  |  | |
| **Yolk** (% of total chorion area, embryos; % of total yolk sac area, larvae) | Control | 82.03 ± 1.15 | 87.65 ± 0.16 | | | 81.60 ± 2.72 | 36.50 ± 6.45 | |
|  | Low | 82.79 ± 0.28 | 87.74 ± 0.56 | | | 78.52 ± 2.19 | 21.60 ± 5.75 | |
|  | Medium | 82.66 ± 0.44 | 86.97 ± 0.51 | | | 77.52 ± 2.15 | 28.63 ± 3.35 | |
|  | High | 82.77 ± 0.61 | 88.11 ± 0.24 | | | 77.90 ± 2.95 | 24.17 ± 4.27 | |
| **Abnormalities** (%) | Control |  |  | | |  | 19.5 ± 2.3 | |
|  | Low |  |  | | |  | 16.6 ± 6.7 | |
|  | Medium |  |  | | |  | 20.8 ± 5.3 | |
|  | High |  |  | | |  | 10.3 ± 1.6 | |
| **Total length** (mm) | Control | 1.22 ± 0.01 | 2.27 ± 0.05 | | | 3.89 ± 0.17 | 4.38 ± 0.03 | |
|  | Low | 1.23 ± 0.01 | 2.21 ± 0.02 | | | 4.15 ± 0.05 | 4.40 ± 0.10 | |
|  | Medium | 1.12 ± 0.01 | 2.25 ± 0.01 | | | 4.11 ± 0.11 | 4.44 ± 0.03 | |
|  | High | 1.21 ± 0.01 | 2.30 ± 0.03 | | | 4.15 ± 0.13 | 4.18 ± 0.11 | |
| **Spine length** (mm) | Control |  |  | | | 3.29 ± 0.14 | 3.86 ± 0.29 | |
|  | Low |  |  | | | 3.52 ± 0.07 | 3.59 ± 0.06 | |
|  | Medium |  |  | | | 3.39 ± 0.04 | 4.23 ± 0.53 | |
|  | High |  |  | | | 3.42 ± 0.06 | 3.43 ± 0.12 | |
| **Head width** (mm) | Control | 0.40 ± 0.01 | 0.50 ± 0.00 | | | 0.55 ± 0.00 | 0.59 ± 0.01 | |
|  | Low | 0.40 ± 0.01 | 0.50 ± 0.00 | | | 0.56 ± 0.00 | 0.63 ± 0.01 | |
|  | Medium | 0.39 ± 0.01 | 0.50 ± 0.00 | | | 0.57 ± 0.01 | 0.60 ± 0.02 | |
|  | High | 0.39 ± 0.01 | 0.50 ± 0.00 | | | 0.60 ± 0.01 | 0.60 ± 0.02 | |
| **Eye diameter** (mm) | Control | 0.22 ± 0.00 | 0.26 ± 0.00 | | | 0.26 ± 0.00 | 0.28 ± 0.01 | |
|  | Low | 0.22 ± 0.00 | 0.26 ± 0.00 | | | 0.27 ± 0.00 | 0.30 ± 0.00 | |
|  | Medium | 0.23 ± 0.01 | 0.26 ± 0.00 | | | 0.27 ± 0.00 | 0.29 ± 0.00 | |
|  | High | 0.22 ± 0.00 | 0.30 ± 0.00* | | | 0.30 ± 0.01 | 0.30 ± 0.01 | |
| **Pupil diameter** (mm) | Control |  | 0.10 ± 0.00 | | |  |  | |
|  | Low |  | 0.09 ± 0.00 | | |  |  | |
|  | Medium |  | 0.10 ± 0.00 | | |  |  | |
|  | High |  | 0.10 ± 0.00 | | |  |  | |
| **Heart beat** (beats/min) | Control |  | 26.19 ± 4.73 | | |  | 35.62 ± 4.27 | |
|  | Low |  | 22.89 ± 2.49 | | |  | 27.55 ± 1.60 | |
|  | Medium |  | 19.51 ± 2.11 | | |  | 28.99 ± 0.28 | |
|  | High |  | 20.77 ± 1.86 | | |  | 30.66 ± 2.90 | |


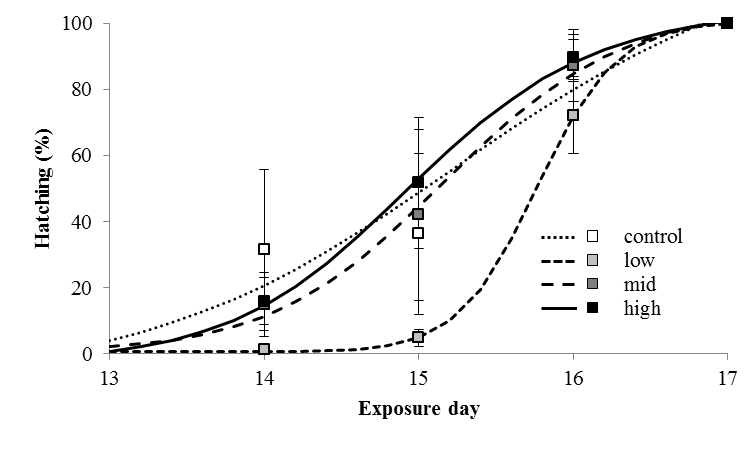


**Figure S1:** Timing of larval hatching. Data are percentage means ± s.e.m., n=3 aquaria per treatment. Modelled with 4 parameter logistic regression ($y=y_{0}+ \frac{a}{1+ \left( \frac{x}{x_{0}} \right)^{b}}$), p < 0.05 model fit; no difference between models from different treatments, GLM F-ratio 141.87, p > 0.05.

**References**

Caipang, C.M.A.; Lazado, C.C.; Brinchmann, M.F.; Rombout, J.H.W.M; Kiron, V., Differential expression of immune and stress genes in the skin of Atlantic cod (*Gadus morhua*). *Comparative Biochemistry and Physiology* **2011,** *Part D 6*, 158-162.

Giannetti, A.; Nagasawa, K.; Fasulo, S.; Fernandes, J.M.O., Influence of photoperiod on expression of *DNA (cytosine-5) methyltransferases* in Atlantic cod. *Gene* **2013,** *519*, 222-230.

Holen, E.; Olsvik, P.A., β-naphthoflavone interferes with *cyp1c1*, *cox2* and *IL-8* gene transcription and leukotriene B_4_ secretion in Atlantic cod (*Gadus morhua*) head kidney cells during inflammation. *Fish & Shellfish Immunology* **2016,** *54*, 128-134.

Nagasawa, K.; Giannetto, A.; Fernandes, J. M. O., Photoperiod influences growth and mll (mixed-lineage leukaemia) expression in Atlantic cod. *PLOS ONE* **2012,** *7*, (5), e36908.

Olsvik, P. A.; Amlund, H.; Sæle, Ø.; Ellingsen, S.; Skjaerven, K. H., Impact of dietary selenium on methylmercury toxicity in juvenile Atlantic cod: A transcriptional survey. *Chemosphere* **2015,** *120*, 199-205.

Olsvik, P.A.; Søfteland, L.; Lie, K.K., Selection of reference genes for qRT-PCR examination of wild populations of Atlantic cod *Gadus morhua*. *BMC Research Notes* **2008,** *1:47.*

Penglase, S.; Hamre, K.; Olsvik, P.A,; Grøtan, E.; Nordgreen, A. Rotifers enriched with iodine, copper and manganese had no effect on larval cod (*Gadus morhua*) growth, mineral status or redox system gene mRNA levels. *Aquaculture Research* **2015,** *46,* 1793-1800.

Skjærven, K. H.; Hamre, K.; Penglase, S.; Finn, R. N.; Olsvik, P. A., Thermal stress alters expression of genes involved in one carbon and DNA methylation pathways in Atlantic cod embryos. *Comparative Biochemistry and Physiology Part A: Molecular & Integrative Physiology* **2014,** *173*, (Supplement C), 17-27.
